# Faricimab Effectively Resolves Intraretinal Fluid and Preserves Vision in Refractory, Recalcitrant, and Nonresponsive Neovascular Age-Related Macular Degeneration

**DOI:** 10.7759/cureus.40100

**Published:** 2023-06-07

**Authors:** Anny M Cheng, Sunir Joshi, Raphael G Banoub, Jackson Saddemi, Kakarla V Chalam

**Affiliations:** 1 Ophthalmology, Broward Health, Fort Lauerdale, USA; 2 Ophthalmology, Specialty Retina Center, Coral Springs, USA; 3 Ophthalmology, Florida International University, Herbert Wertheim College of Medicine, Miami, USA; 4 Ophthalmology, South Florida Vision, Fort Lauerdale, USA; 5 Ophthalmology, Loma Linda University School of Medicine, Loma Linda, USA

**Keywords:** central subfield thickness, vabysmo, subretinal fluid, refractory namd, intravitreal anti-vascular endothelial growth factor, intraretinal fluid, faricimab

## Abstract

Purpose: To evaluate the functional and anatomic outcomes of faricimab treatment in patients with neovascular age-related macular degeneration (nAMD) who are unresponsive to other anti-vascular endothelial growth factor (VEGF) therapies.

Methods: A retrospective interventional study was conducted on patients with refractory nAMD who were initially treated with intravitreal bevacizumab, ranibizumab, or aflibercept. These patients were switched to monthly faricimab injections. The central subfield thickness (CST), intraretinal fluid (IRF) or subretinal fluid (SRF) height, and visual acuities were compared before and after faricimab treatment.

Results: A total of 13 eyes (eight right eyes and five left eyes) from 11 patients were followed for 10.4 ± 6.9 months after bevacizumab treatment and 40.3 ± 28.7 months after aflibercept treatment before switching to faricimab. The follow-up time for patients receiving a mean number of 3.7 ± 1.3 faricimab injections was 3.4 ± 1.2 months. The overall median CST was reduced by 18µm (p=0.001) from 342µm to 318µm, along with a reduction of 89µm (p=0.03) in IRF/SRF height from 97µm to 40µm. Following three consecutive injections, the CST showed a significant reduction of 21.5µm (p=0.004) from 344µm to 322.5µm, and IRF/SRF height was reduced by 89µm (p=0.03) from 104µm to 18.5µm. The intraretinal fluid size decreased and leakage stopped, as seen on fluorescein angiography. Visual acuity remained stable after switching to faricimab treatment (0.59 ± 0.45 logMAR vs 0.58 ± 0.45 logMAR, p=1).

Conclusions: Faricimab has proven to be an effective treatment for nAMD patients resistant to other anti-VEGF agents. It demonstrates significant anatomical improvement and vision preservation in this challenging patient population.

## Introduction

Neovascular age-related macular degeneration (nAMD) remains a major cause of severe visual impairment globally, despite the availability of effective therapies such as intravitreal anti-vascular endothelial growth factor (VEGF) agents. In nAMD, VEGF-mediated angiogenesis results in the growth of pathological choroidal neovascularization (CNV) and exudation of blood or serous fluid into the macula, potentially leading to submacular fibrosis and permanent loss of central vision [1,2].

Three anti-VEGF agents (bevacizumab, ranibizumab, and brolucizumab) inhibit all biologically active forms of VEGF, whereas aflibercept functions as a decoy receptor that binds VEGF A, VEGF B, and placental growth factor (PGF) [3]. However, anti-VEGF drugs only target pathological leakage from CNV lesions and do not address other nAMD pathology mechanisms.

The CATT trial showed that 67.4% of eyes treated with monthly bevacizumab injections and 51.5% of eyes treated with monthly ranibizumab had persistent fluid after two years [4]. Around 8.1-15% of nAMD patients were non-responders to bevacizumab and ranibizumab [5,6], while 5.2% were non-responders to aflibercept [7]. Different dosing and injection regimens have been shown to achieve equivalent, but not better, visual acuity than ranibizumab monotherapy after 52 [8] and 96 weeks [9]. Despite monthly ranibizumab injections over 12 or 24 months, 60% and 65% of patients did not achieve 20/40 or better vision, respectively [10]. A separate trial noted that nearly half of the eyes receiving anti-VEGF therapy developed submacular fibrosis, limiting further visual benefits despite extended treatment [11]. Increased dosage and frequency of ranibizumab monotherapy did not demonstrate any additional benefits [12]. The upregulation of other proangiogenic biomarkers, such as angiopoietin-2 and VEGF-C [13], has been observed after bevacizumab injections in patients with nAMD, indicating that one form of VEGF may interact with other proangiogenic factors and limit therapeutic outcomes. 

Elevated intravitreal concentrations of angiopoietin-2 (Ang-2) have been observed in various vascular diseases [14,15], including nAMD [14], and Ang-2 is a promising new target for treating pathological vascular diseases. Unlike traditional monospecific antibodies, faricimab is composed of bispecific heterodimeric antibodies with different light chains in each fragment antigen-binding region, allowing it to bind to two different targets simultaneously. This specific structure of faricimab, which includes a VEGF-binding domain and an Ang-2-binding domain, enables simultaneous and independent binding of VEGF-A and Ang-2, resulting in effective function [16,17]. Faricimab inhibits both VEGF-A and Ang-2 [18], and phase II and III data support the hypothesis that dual-pathway inhibition improves visual and anatomical outcomes in patients with nAMD [19,20]. 

However, the short-term therapeutic effects of faricimab as salvage therapy for nAMD patients who respond poorly or incompletely to other anti-VEGF agents have not been studied. This study specifically reports the short-term therapeutic effects of faricimab in treatment-refractory nAMD patients.

## Materials and methods

This retrospective non-comparative study aimed to evaluate the functional and anatomic outcomes of intravitreal faricimab injection in patients with choroidal neovascularization (CNV) secondary to nAMD who were refractory to intravitreal treatment with bevacizumab, ranibizumab, or aflibercept. The study was conducted between March 2022 and September 2022, and the medical records of patients treated with intravitreal anti-VEGF agents for nAMD were retrospectively reviewed. The study was approved by the Institutional Review Board (Pearl IRB, 22-ARES-101, date of approval:11/14/2022) and conducted in accordance with the Declaration of Helsinki. All patients provided informed consent for the treatment and their potential side effects.

Patients who demonstrated inadequate response to bevacizumab, ranibizumab, or aflibercept were switched to faricimab injections administered monthly by an experienced retina specialist (SJ). The inclusion criteria for the study comprised patients with refractory nAMD who were initially treated with bevacizumab or ranibizumab and later switched to aflibercept and received a minimum of three injections of aflibercept. An inadequate response was defined as persistent, worsening, or recurrent intraretinal fluid (IRF) or subretinal fluid (SRF) on spectral-domain optical coherence tomography (SD-OCT) scans after three consecutive intravitreal injections. The exclusion criteria were CNV secondary to causes other than nAMD; concurrent retinal vascular disorders; and continuous small improvement to bevacizumab, ranibizumab, or aflibercept with residual IRF or SRF, or good response in cases of recurrence.

At each follow-up visit, patients underwent Snellen visual acuity testing, fundoscopic examination, and SD-OCT. The primary outcome of the study was the assessment of anatomical changes through the measurement of CST and IRF or SRF height using SD-OCT (Zeiss Cirrus, Carl Zeiss Meditec, Germany). Secondary outcomes were changes in visual acuity (logMAR) and adverse effects. The foveal thickness was determined by the clinician (AC) as the distance between the internal limiting membrane and outer Bruch's membrane at the level of the fovea. Initial IRF or SRF height was defined as the vertical distance between the largest hyporeflective space above the outer nuclear layer or sensory retina and the inner edge of the retinal pigment epithelium (RPE). If multiple IRFs or SRFs were present, the maximum height was selected. Changes in IRF or SRF height were measured along the same meridian before and after treatment.

Statistical analysis was performed using a two-sample, two-tailed paired t-test with SPSS statistical software V. 22.0. IBM Corp, Armonk, NY). Descriptive statistics are presented as mean ± standard deviation or median (interquartile range: IQR) if they did not follow a normal distribution. Pearson's correlation analysis was used to show the linear correlation between the variables, and statistical significance was set at P < 0.05.

## Results

A total of 13 eyes from 11 patients, comprising four men (36%) and seven women (64%), were analyzed. The mean age of the patients was 81.8 ± 6.7 years (Table 1). Of the 13 eyes, one eye (7.7%) was initially treated with aflibercept, while the remaining 12 eyes (92.3%) were initially treated with bevacizumab. Of the 12 eyes treated with bevacizumab, 11 (92%) were switched to aflibercept because of persistent, worsening, or recurrent IRF/SRF. The remaining eyes (8%) switched to ranibizumab and then to aflibercept because of poor treatment response. Most patients had a long follow-up period before switching to faricimab, with a mean follow-up time of 49.9 ± 25.3 months. The mean follow-up time from bevacizumab to aflibercept was 10.4 ± 6.9 months and from aflibercept to faricimab was 40.3 ± 28.7 months.

**Table 1:** Patient demographics and faricimab treatment results Abbreviations: BCVA: best corrected visual acuity; CST: central subfield thickness; diff: difference; F: female; fu: follow up; IRF: inj: injection; intraretinal fluid; logMAR: Log of Minimum Angle of Resolution; M: male; mths: months; No: number; OD: right eye; OS: left eye; SRF: subretinal fluid;tx: treatment; yrs: years; µm: micrometers

Case No	Gender/ age(yrs)/ eye	No. of Inj	CST before faricimab tx(µm)	CST 1 mth diff (µm)	CST 3 mths diff (µm)	CST overall diff (µm)	IRF/SRF Height after faricimab tx(µm)	IRF/SRF Height 1 mth diff(µm)	IRF/SRF Height 3 mths diff(µm)	IRF/SRF Height overall diff(µm)	BCVA baseline (logMAR)	BCVA after faricimab tx (logMAR)	BCVA diff (logMAR)	faricimab tx fu (mths)
1	F/80/OD	1	464	0	-9	-9	422	-111	-124	-155	0.544	0.544	0	4.9
		2	464				311							
		3	406				304							
		4	455				298							
		5	455				267							
2	M/89/OD	1	455	-142	-208	-196	215	-171	-215	-215	0.398	0.398	0	4.8
		2	313				44							
		3	360				37							
		4	247				0							
		5	265				0							
		6	259				0							
3	F/87/OD	1	346	-1	-22	-31	67	75	-67	-67	0.602	0.602	0	3.7
		2	345				142							
		3	323				0							
		4	324				0							
		5	315				0							
4	F/72/OS	1	416	-56	-80	-80	111	-111	-111	-111	1.301	1.301	0	3.7
		2	360				0							
		3	327				0							
		4	336				0							
5	M/84/OD	1	369	28	-48	-48	97	-1	-53	-53	0.544	0.544	0	3.7
		2	397				96							
		3	386				52							
		4	321				44							
6	M/84/OS	1	306	-13	-21	-21	89	-89	-89	-89	0.176	0.176	0	2.6
		2	293				0							
		3	285				0							
7	M/77/OS	1	326	-41	-49	-49	252	-119	-133	-133	1	1	0	2.8
		2	285				133							
		3	277				119							
8	F/82/OS	1	342	-2	-10	-10	67	-15	-30	-30	0.301	0.301	0	2.8
		2	340				52							
		3	337				52							
		4	332				37							
9	F/89/OD	1	248	-5	-2	-4	682	-15	-177	-238	0.301	0.301	0	6.1
		2	243				667							
		3	245				563							
		4	246				505							
		5	244				444							
10	M/87/OD	1	314	4	-	4	74	-34	-	-34	1.602	1.602	0	1.9
		2	318				40							
11	F/69/OD	1	340	2	2	2	89	-15	-89	-89	0.0969	0.0969	0	2.8
		2	342				74							
		3	342				0							
12	F/69/OD	1	315	-8	-	-8	74	-13	-	-13	0.301	0.301	0	2.7
		2	307				61							
13	F/84/OD	1	521	-18	-	-18	259	52	-	52	0.544	0.398	-0.146	1.6
		2	503				311							
median (µm)				-5	-21.5	-18		-15	-89	-89				
Interquartile range (µm)				1 to 29.5	7.25 to 56.8	6 to 48.5		7 to 111	70.5 to 155	32 to 144				

Reduction of CST, IRF/SRF height, and cessation of leakage on fluorescein angiography with faricimab

**Figure 1:**
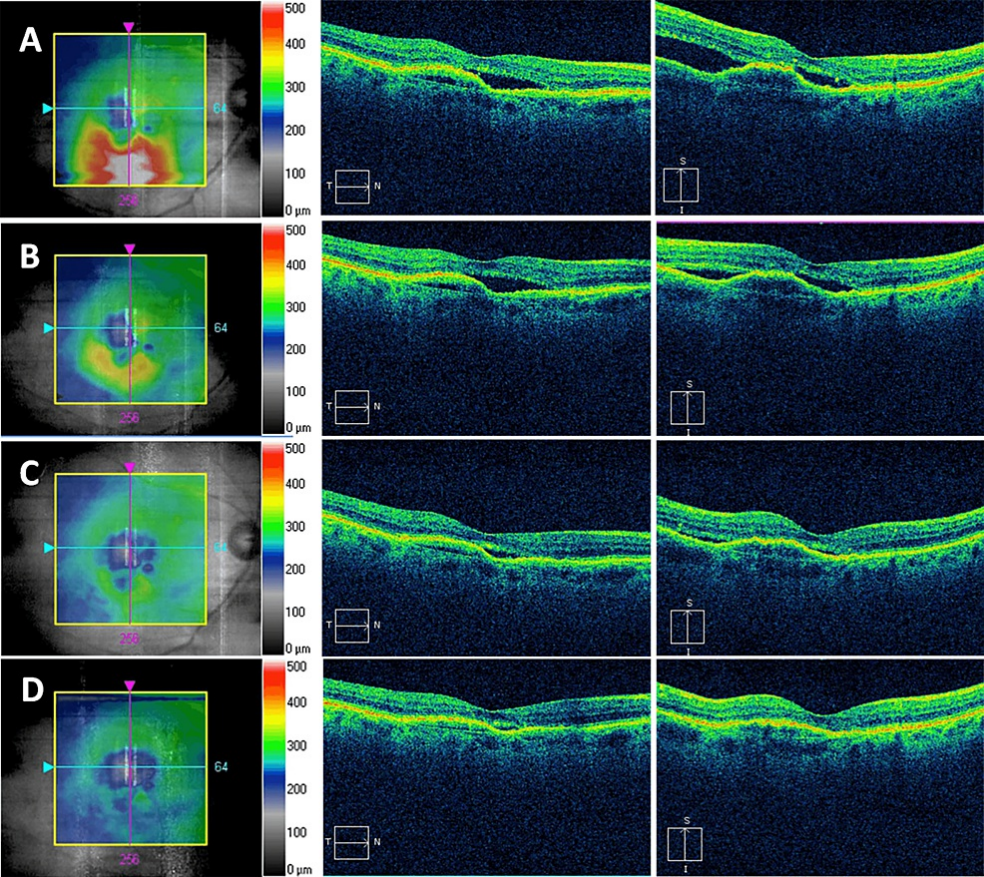
Faricimab in a patient with refractory neovascular age-related macular degeneration An 89-year-old man with 12 months on bevacizumab followed by 38 months of aflibercept treatment showed persistent subretinal fluid (SRF) in the right eye (A). Optical coherence tomography images one month (B), two months (C), and four months (D) after monthly faricimab injection showed a reduction of central subfield thickness and resolution of SRF.

**Figure 2:**
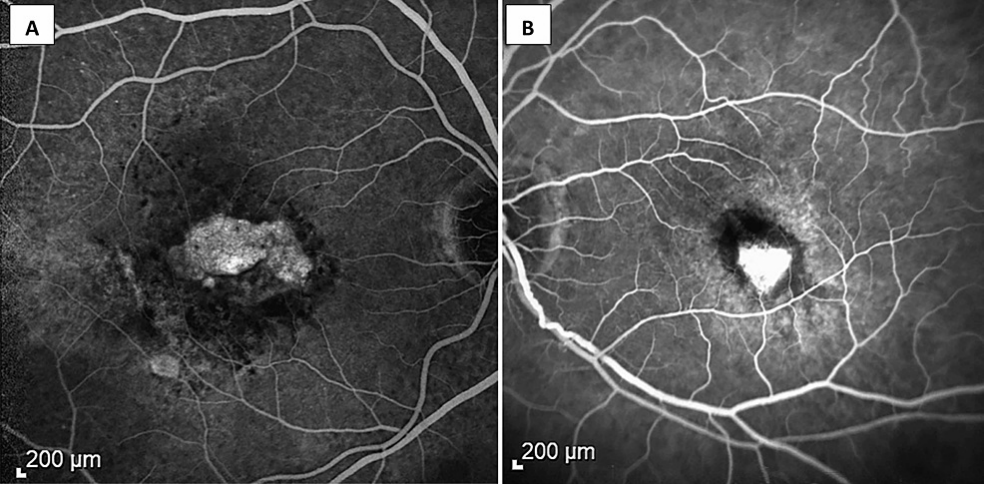
Absence of leakage after treatment with farcimab in recalcitrant AMD on fluorescein angiogram in two patients (A, B). (A) Resolution of edema with staining of the neovascular membrane. (B) Intense staining of the regressed membrane after Faricimab therapy.

Reduction in CST, IRF/SRF height, and cessation of leakage were observed with faricimab treatment. The mean follow-up time for patients receiving faricimab was 3.4 ± 1.2 months, and the overall median CST was reduced by 18 µm (IQR 6 µm to 48.5 µm, p = 0.001) from 342 µm (IQR 314.5 µm to 435.5 µm) to 318 µm (IQR 281 µm to 339 µm) along with a reduction of 89 µm (IQR 32 µm to 144 µm, p = 0.03) in IRF/SRF height from 97 µm (IQR 74 µm to 255.5 µm) to 40 µm (IQR 0 to 163.8 µm) (Table 1). After one month and one injection of faricimab, the median CST reduced by 5 µm (IQR 1 µm to 29.5 µm, p = 0.064), and the IRF/SRF height reduced by 15 µm (IQR 7 µm to 111 µm, p = 0.2). After three consecutive injections (n = 10), the median CST significantly reduced from 344 µm (IQR 321 µm to 425.8 µm) to 322.5 µm (IQR 269.5 µm to 337.5 µm) with an improvement of 21.5 µm (IQR 7.25 µm to 56.8 µm, p = 0.004) (Figure 1), and the IRF/SRF height significantly reduced by 89 µm (IQR 70.5 µm to 155 µm, p = 0.03) from 104 µm (IQR 83.5 µm to 294.5 µm) to 18.5 µm (IQR 0 to 163.8 µm). In addition, there was a decrease in intraretinal fluid size and cessation of leakage on fluorescein angiography (Figure 2).

Stable visual acuity with faricimab

The mean best corrected visual acuity (BCVA) remained stable with faricimab treatment, with a mean BCVA of 0.59 ± 0.45 logMAR prior to switching to faricimab and 0.58 ± 0.45 logMAR at the final examination after initiation of faricimab treatment (p = 1). Only one of the 13 eyes (7.7%) showed an improvement of two lines.

No correlation was observed between the number of previous injections and the decrease in CST (r = 0.001, p = 0.98). No instances of ocular endophthalmitis or systemic side effects were reported.

## Discussion

The present study quantifies the anatomical improvement obtained with faricimab treatment for refractory cases of nAMD. Clinical improvement with faricimab in Phase II was comparable to that of ranibizumab monotherapy [20]. The results of two Phase III randomized, double-masked clinical trials (TENAYA and LUCERNE) showed non-inferior visual improvement with 6 mg faricimab dosed every three or four months compared to aflibercept monotherapy in 34% and 45.7% (TENAYA) and 32.9% and 44.9% (LUCERNE) of patients, respectively [21].

Recent clinical studies showed that faricimab has comparable improvements in anatomical parameters, including a reduction in CST, and a reduction in the size of CNV lesions in treatment-naive nAMD patients [22,23]. In a Japanese study of 63 treatment-naive eyes with nAMD, complete resolution of CST and IRF/SRF was observed in 82% of the eyes after three consecutive monthly intravitreal injections of faricimab [22]. Similarly, in another Japanese study of 40 treatment-naive eyes with monthly faricimab injections, CST reduced significantly and IRF/SRF resolved completely in 79.5% of eyes at the 16-week follow-up [23].

Despite the demonstrated effectiveness of faricimab in treatment-naive nAMD patients, the therapeutic effects of faricimab in refractory nAMD patients remain unclear [21]. Our study showed that CST and IRF/SRF were reduced after the first faricimab treatment; however, the difference was not statistically significant. After receiving at least three consecutive faricimab injections, refractory nAMD patients with an inadequate or diminished response to other anti-VEGF injections showed statistically significant reductions in CST and IRF/SRF height, while maintaining stable visual acuity. Similar findings were described in an American study that included multicenter patients with previously treated (n=337) or treatment-naive (n = 39) eyes. Overall, CST reduction was found in all eyes after one injection (n=376) and three injections (n=94) [24]. Our results were also comparable to a non-hospital-based study of 190 eyes with previous treatment-resistant nAMD. The CST improved significantly after receiving approximately monthly (5.16±2.8 weeks) faricimab injections with an average of 34.88±8.2 weeks of follow-up [25].

The improved response to faricimab in previously unresponsive patients may be attributed to its pharmacological targets. Combination therapy with anti-VEGF plus anti-platelet-derived growth factor (PDGF) [26] or anti-placental growth factor (PGF) [27,28] have been proposed; however, two-thirds of patients failed to gain three or more lines after 52 weeks of combination therapy with aflibercept and anti-PGF [8]. Some investigational combination anti-PDGF and anti-VEGF drugs demonstrated favorable visual acuity outcomes compared to anti-VEGF monotherapy [29], but OCT did not reveal anatomical differences between the groups in a phase III trial. Studies investigating the treatment efficacy of combined anti-PDGF and anti-VEGF agents for nAMD were terminated [26]. The Ang/Tie-2 pathway is a promising therapeutic target for retinal vascular diseases [30], and preclinical studies have shown that dual inhibition of the Ang/Tie pathway and VEGF-A yields better outcomes than anti-VEGF monotherapy alone [31,32].

Resistance to anti-VEGF agents has been identified in animal and clinical studies, with resistance to ranibizumab, detected in 11.1% of patients receiving 10 or fewer injections and 21.7% of patients receiving more than 10 intravitreal injections [33]. A single intravitreal injection of aflibercept and ranibizumab in monkeys resulted in immune reactivity against these anti-VEGF drugs [34], suggesting that an immune response may lower their therapeutic effects. This may explain the improved response observed after switching from a pure anti-VEGF agent to a dual-mechanism agent, such as faricimab.

In our study, faricimab demonstrated significant anatomical advancements; however, it did not result in visual improvement. This could be attributed to the selection of patients who had severe and treatment-resistant diseases; thus, their visual function might have been restricted due to morphological and anatomical limitations that were not considered in the patient selection process. This study had certain limitations, such as its retrospective design, limited sample size, and brief treatment and follow-up periods. The effectiveness of faricimab with extended treatment intervals of three to four months in patients with refractory nAMD, similar to the patients in our study, is still uncertain. Therefore, further large-scale, prospective, and controlled trials are necessary to determine the optimal therapeutic role of faricimab in the treatment of refractory nAMD.

## Conclusions

In conclusion, our study found promising results with the use of faricimab, a newly introduced agent, in treating patients with refractory nAMD. The use of faricimab resulted in significant improvements in anatomical outcomes and the preservation of visual acuity in patients who had previously not responded to other treatments. These results indicate that faricimab could be an effective treatment option for eye care providers to consider when administering intravitreal injections to patients with refractory nAMD.
